# Resource Allocation in Unmanned Aerial Vehicle (UAV)-Assisted Wireless-Powered Internet of Things

**DOI:** 10.3390/s19081908

**Published:** 2019-04-22

**Authors:** Bingjie Liu, Haitao Xu, Xianwei Zhou

**Affiliations:** School of Computer and Communication Engineering, University of Science and Technology Beijing, Beijing 100083, China; b20160290@xs.ustb.edu.cn (B.L.); xwzhouli@sina.com (X.Z.)

**Keywords:** resource allocation, wireless power transfer, UAV, Internet of Things, dynamic game

## Abstract

Most of the wireless nodes in the Internet of Things (IoT) environment face the limited energy problem and the way to provide a sustainable energy for these nodes has become an urgent challenge. In this paper, we present an unmanned aerial vehicle (UAV) to power the wireless nodes in the IoT and an investigation on the optimal resource allocation approach based on dynamic game theory. This IoT system consists of one UAV as the power source and information receiver. The wireless nodes can be powered and collected by the UAV. In order to distinguish the wireless nodes, the wireless nodes are divided into two categories based on the energy consumption. The UAV tries to power these two categories of nodes using a different power level based on the proposed approach, where the wireless nodes control the resources for information transmission. Based on Bellman dynamic programming, the optimal allocated resources for power transfer and information transmission are obtained for both the UAV and wireless nodes, respectively. In order to show the effectiveness of the proposed model and approach, we present numerical simulations.

## 1. Introduction

The Internet of Things (IoT) [1,2,3,4] is playing an important and significant role in the next generation mobile communication and wireless networks and is currently used in various services in our daily life. With the implementation of the Internet of Everything, data transmission has become denser, and the amount of information exchange is becoming larger and larger. Therefore, it is essential to use new communication technologies with a greater bandwidth, higher speed, lower latency, and lower energy consumption, to guarantee the popularity of the IoT [5,6]. Meanwhile, due to the limited energy of the wireless devices in the IoT, the developments and applications of IoT technologies are facing unprecedented and severe challenges. How to provide sustainable energy for the wireless devices in IoT has become an urgent problem to be solved, restricting the development of IoT.

As an important method for sustainable energy supply, wireless power transfer (WPT) technology is regarded as an ideal solution for providing sustainable energy to the wireless devices in the IoT and can effectively solve the bottleneck of the limited energy problem in IoT [7,8]. More and more wireless devices may use WPT technology to reduce the excessive dependence on the battery. WPT has been widely used in portable electronic devices, implanted medical devices, smart homes, electric vehicles (EVs), and so on [9]. Applying the WPT to wireless communication networks can permit wireless powered communication networks (WPCN) to be constructed and can effectively improve the energy efficiency of the network [10,11,12,13,14,15,16,17,18,19]. Compared with the conventional WPT system with a relatively fixed energy source, UAV can dynamically move the IoT devices around, power the IoT devices, and realize the data gathering and transmitting services [20]. The UAV-assisted WPT can greatly improve the performance of the WPCN by dynamically adjusting the power source position. Utilizing the high mobility of UAV, the WPCN can provide ubiquitous energy for wireless devices with a large area distribution, which is faster, more flexible, and more controllable.

In this paper, we research the UAV-assisted wireless-powered IoT and solve the resource allocation problem in the proposed IoT system. The resource allocation problem between the UAV and wireless nodes is researched based on a dynamic game and dynamic programming. In our proposed system, the wireless nodes harvest energy from the UAV and transmit data to the UAV. The UAV works as a power source that can move around to charge the wireless nodes based on the wireless power transfer technique. The UAV can also gather all the data from the wireless nodes. A dynamic game-based model for the resource allocation problem between the UAV and wireless nodes is given, and Nash equilibriums for the model can be obtained based on Bellman dynamic programming. Based on the Nash equilibriums, the UAV can optimally allocate its energy resources for wireless power transfer. The main innovations and contributions are summarized as follows:

- Firstly, the UAV-assisted wireless-powered IoT system is given, including one UAV and many wireless nodes. The UAV tries to harvest the wireless nodes based on the wireless energy transfer techniques. The wireless nodes use the harvested energy for information transmission;
- Secondly, the wireless nodes are divided into two categories based on their energy consumption. The wireless nodes with different energy consumptions will have different energy supplied from the UAV. The energy transferred from the UAV should be different based on the energy consumption;
- The resource allocation problem between the UAV and wireless nodes is formulated as a dynamic game. In the proposed dynamic game, the UAV can optimally control its resources for energy transfer, and the wireless nodes can optimally control their resources for information transmission;
- Finally, based on the dynamic game, we have proposed two objective functions for the UAV, which are both formulated as profit maximization functions. The objective functions will be distinguished for different wireless nodes based on the energy consumption assumptions.

The remainder of the paper is organized as follows: Section 2 summarizes the related work in WPT and UAV assisted WPT. The system model and problem formulation are given in Section 3. The analysis of the proposed game model is shown in Section 4. The numerical simulations are given in Section 5, and finally, we conclude the work in Section 6. 

## 2. Related Work

The WPT technique, as an essential way to solve the limited energy problem in IoT, has been researched by many academics. In reference [10], an energy efficient power management approach is proposed according to the user’s Quality of Services (QoS) requirements in WPCN, and the optimal power of the power stations can be obtained based on the authors’ adaptation algorithm. In [11], multiple users can obtain energy from fixed power stations in WPCN, and through the combination of power control and time allocation, the problem of network energy efficiency maximization is studied. From the above references, we can view that in the conventional WPT system, the energy source is relatively fixed in position. Recently, an unmanned aerial vehicle (UAV) has been used in many applications, such as traffic monitoring [12], data transmission [13], communication enhancement [14,15], and so on. In order to solve the fixed energy source problem in the conventional WPT system, UAV can be used due to its inherent agility and on-demand quality [16], which has attracted considerable attention in the IoT [17,18,19,20]. 

In order to supply removable energy, UAV is used as new kind of power source for IoT devices [21,22,23,24,25,26]. References [21,22] have studied the resource allocation problem of the UAV auxiliary network. Using UAV as the energy source, this network can provide energy and information collection services for the devices and maximize the transmit rate to improve the system throughput. Zeng et al. [23] have established an energy consumption model of the UAV system to solve the energy-efficient communication problem via optimizing the trajectory of UAV, and have improved the energy utilization rate of the UAV system. In [24], the relay function was installed on the UAV, and an approach was proposed to optimize the throughput in the UAV-based relay system. The transmit power and relay trajectory were optimized in this paper. Zhang et al. [25] studied a mobile relay system that supports UAV to achieve tradeoff between the maximum spectrum efficiency and bits/Joule energy efficiency by taking advantage of the new degree of freedom of UAV trajectory design.

## 3. System Model and Problem Formulation

**Figure 1:**
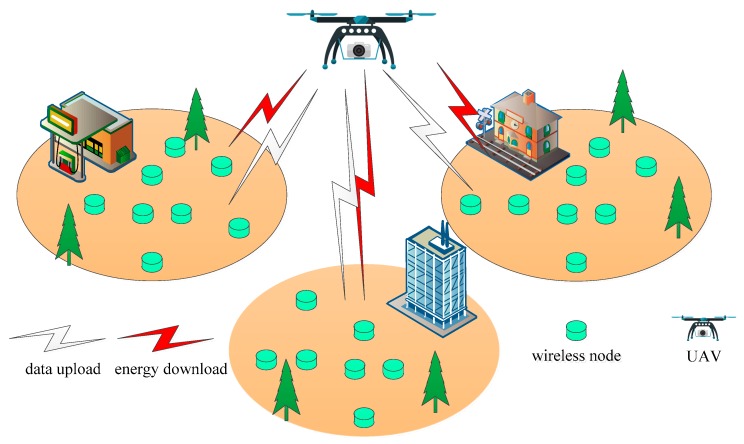
System model. UAV, unmanned aerial vehicle.

As shown in Figure 1, a UAV-assisted wireless-powered IoT system is proposed, which includes a power source UAV and a large number of wireless nodes [26]. The UAV is assumed to move around the wireless nodes for wireless power transfer, and the wireless nodes need harvest energy from the UAV, and use the harvested energy to transmit information. Our goal is to find the optimal resource allocation approaches for the wireless power transfer and information transmission in the proposed IoT system. In our proposed system, the wireless nodes require energy from the UAV, and transmit information to the UAV using the received energy. The UAV works as the power source that moves around to charge the wireless nodes. The UAV can also gather all the information from the wireless nodes. In this paper, although the wireless nodes are randomly distributed in the IoT environment, they are all assumed to be charged by the UAV.

**Figure 2:**
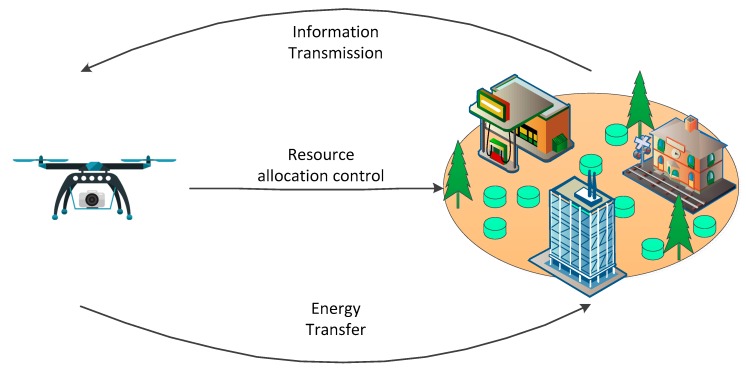
Leader-follower relationships between the UAV and the wireless nodes.

In the IoT system, different wireless nodes will be allocated different tasks for data collection and transmission, and the energy consumptions of the wireless nodes should be different. Based on this assumption, in order to distinguish the various wireless nodes, we divide the wireless nodes into two categories. One includes the higher energy consumption nodes (HEC nodes), which need more energy for data collection and transmission, and the other includes the lower energy consumption nodes (LEC nodes), with lower energy requirements. Then, the energy transferred to the wireless nodes from the UAV will be different. The HEC nodes may have more energy transferred from the UAV compared with the LEC nodes. During the data transmission, the wireless nodes in the same energy consumption category are assumed to share the same channel, and will cause inter-channel interferences. It is assumed that when the UAV transfers energy to the wireless nodes in one category, there is no energy transfer and information transmission between the UAV and the wireless nodes in the other category. Then, the intra-channel interferences between the two categories can be ignored. The aim of the paper is to find the optimal resource allocation solutions for the UAVs to harvest the wireless nodes, and for the wireless nodes to transmit information. It is assumed there are *N_H_* nodes in the HEC category, and ${Ν}_{H}=\left[1_{H},\ldots ,N_{H}\right]$ is the HEC nodes set. The number of the nodes in the LEC category is denoted by *N_L_*, and the LEC nodes set is ${Ν}_{L}=\left[1_{L},\ldots ,N_{L}\right]$. Because the wireless nodes use the harvested energy for information transmission, the wireless nodes should pay for the UAV for energy harvesting and the resources for information transmission are affected by the available harvested energy. UAV can control the power level for energy transfer and can control the unit energy price for the wireless nodes. Based on all these assumptions given in the above, the UAV is considered to be the leader of the dynamic game, and the wireless nodes are treated as the followers. The relationships between the UAV and the wireless nodes are shown in Figure 2.

Let $p_{H,i}\left(t\right)\left(i\in \left\{{Ν}_{H}\right\}\right)$ denote the allocated resource of the HEC nodes, and $p_{L,i}\left(t\right)\left(i\in \left\{{Ν}_{L}\right\}\right)$ represent the allocated resource of node i in the LEC category. In order to achieve information transmission between the wireless nodes and the UAV, the allocated resources given by $p_{H,i}\left(t\right)\left(i\in \left\{{Ν}_{H}\right\}\right)$ and $p_{L,i}\left(t\right)\left(i\in \left\{{Ν}_{L}\right\}\right)$ mean the minimum energy requirements of the wireless nodes from the UAV. The wireless nodes transmit information to the UAV using the required energy from the UAV. When the wireless nodes obtain the energy form the UAV, they can use the obtained energy to transmit the information and earn revenue from the information transmission process. As the wireless nodes in the same category transmit information in the same channel, there exists inter-cell interference among the wireless nodes in the same category. At this point, we can use the signal to interference noise ratio (SINR) to denote the revenue earned from the information transmission, which can be expressed in the form of the power level for information transmission as follows:

(1) $$\gamma_{k,i}=\frac{g_{k,i}\left(t\right)p_{k,i}\left(t\right)}{\sum_{j\neq i}g_{k,j}^{k,i}\left(t\right)p_{k,j}\left(t\right)+\sigma^{2}\left(t\right)}=\frac{g_{k,i}\left(t\right)p_{k,i}\left(t\right)}{I_{k,i}\left(t\right)}$$

where *k* is the category number and k={H,L}. $g_{k,i}\left(t\right)$ is the channel gain from transmit node *i* in category *k* to the UAV. $\sigma^{2}\left(t\right)$ is the background noise, and $\sum_{j\neq i}g_{k,j}^{k,i}\left(t\right)p_{k,j}\left(t\right)$ is the inter-cell interference from the other transmit node *j* in the category *k* to the transmit node *i*. Then, $I_{k,i}\left(t\right)=\sum_{j\neq i}g_{k,j}^{k,i}\left(t\right)p_{k,j}\left(t\right)+\sigma^{2}\left(t\right)$ is the total interference for the wireless node *i* in category *k*, which is the overall sensed interference by the wireless node *i* in category *k*. It is assumed that the nodes in our proposed system know their distance between the nodes and the UAV, and the distance among the nodes. The effect of distance on transmission is uniformly represented by the channel gain. Based on the SINR definition given in (1), the cost function of energy transfer for the wireless nodes during the observation time [0,T] is given as [27]:

(2) $$\max\int_{0}^{T}\left\{{\left[\gamma_{k,i}\left(p_{k,i}\left(t\right)\right)-\gamma_{th}\right]}^{2}-\pi_{k}\left(t\right)p_{k,i}\left(t\right)\right\}e^{-rt}dt$$

where $\gamma_{th}$ denotes the SINR threshold, which also means the QoS constraint; $\pi_{k}\left(t\right)$ is the unit price of energy transfer controlled by the UAV at time *t*; *r* is the discount rate; and $e^{-rt}$ is the discount factor. Solving the above equation yields the optimal solutions of $p_{k,i}\left(t\right)$:

(3) $$p_{k,i}^{}\left(t\right)=\frac{1}{2}\pi_{k}\left(t\right)\frac{I_{k,i}^{2}\left(t\right)}{g_{k,i}^{2}\left(t\right)}+\gamma_{th}\frac{I_{k,i}^{}\left(t\right)}{g_{k,i}^{}\left(t\right)}$$

The wireless nodes in different categories will enjoy different energy supply services from the UAV, because the energy consumptions are different. Energy transferred to one kind of wireless node cannot be harvested by the other kind of node. Based on the dynamic game, the UAV should make its choice on the power level for energy transfer, and the wireless nodes in different categories should control their power level for information transmission. Let *x*(*t*) denote the state function of the proposed dynamic game model, which is the energy level of the whole IoT system at time *t*. The dynamic variation of the state function can be described by the following differential equation:

(4) $$\frac{dx\left(t\right)}{dt}=\sum_{k=\left\{H,L\right\}}^{}\eta_{k}p_{k}\left(t\right)+\sum_{i=1}^{N_{H}}p_{H,i}\left(t\right)+\sum_{i=1}^{N_{L}}p_{L,i}\left(t\right)+\delta x\left(t\right)$$

where $p_{k}\left(t\right)$ is the allocated power level for energy transfer for the nodes in category *k*; $\eta_{k}$ denotes the energy conversion efficiency; and δ denotes the energy consumption rate of the whole IoT system, which should be a constant value. The initial value of the system state is denoted by $x_{0}=x\left(0\right)$.

In this paper, we have divided the set of wireless nodes into two categories, which are the HEC nodes and the LEC nodes. Each category will be allocated different energy. For the UAV, once the energy is transferred to one kind of wireless node, there will be less energy available for the other kind of wireless node. In this paper, this kind of energy transfer effect is some kind of category of activity, and is considered the energy transfer cost for the system. In our model, the “harvest-then-transmit” protocol [28] is used, and the wireless energy transfer and wireless information transmission can operate at the same frequency. Then, information transmission will cause interferences to the UAV. In this paper, we use $\varepsilon_{H}^{H}$ and $\varepsilon_{H}^{L}$ to denote the information transmission interference of the wireless nodes to the HEC nodes, and use $\varepsilon_{L}^{L}$ and $\varepsilon_{L}^{H}$ to denote the information transmission interference of the wireless nodes to the LEC nodes. The UAV that is responsible for the energy transfer of the two categories of wireless nodes should maximize the energy transfer profit minus the cost during the wireless information transmission and power transfer process. The objective functions of the UAV for the two categories of wireless nodes are given by

(5) $$V^{H}\left(t\right)=\max\int_{0}^{T}\left[\begin{array}{l} \sum_{i=1}^{N_{H}}\left[{\left[\gamma_{H,i}\left(p_{H,i}\left(t\right)\right)-\gamma_{th}\right]}^{2}-\pi_{H}\left(t\right)p_{H,i}\left(t\right)\right]-c_{H}p_{H}^{2}\left(t\right)\\ -\varepsilon_{H}^{H}\sum_{i=1}^{N_{H}}p_{H,i}\left(t\right)-\varepsilon_{H}^{L}\sum_{i=1}^{N_{L}}p_{L,i}\left(t\right)-\mu_{H}x\left(t\right) \end{array}\right]e^{-rt}dt+s_{H}x\left(T\right)e^{-rT}$$

(6) $$V^{L}\left(t\right)=\max\int_{0}^{T}\left[\begin{array}{l} \sum_{i=1}^{N_{L}}\left[{\left[\gamma_{L,i}\left(p_{L,i}\left(t\right)\right)-\gamma_{th}\right]}^{2}-\pi_{L}\left(t\right)p_{L,i}\left(t\right)\right]-c_{L}p_{L}^{2}\left(t\right)\\ -\varepsilon_{L}^{L}\sum_{i=1}^{N_{L}}p_{L,i}\left(t\right)-\varepsilon_{L}^{H}\sum_{i=1}^{N_{H}}p_{H,i}\left(t\right)-\mu_{L}x\left(t\right) \end{array}\right]e^{-rt}dt+s_{L}x\left(T\right)e^{-rT}$$

In (5) and (6), $c_{H}p_{H}^{2}\left(t\right)$ denotes the cost of energy transfer to the HEC nodes, and $c_{L}p_{L}^{2}\left(t\right)$ denotes the cost of energy transfer to the LEC nodes. $\mu_{H}$ and $\mu_{L}$ are the natural energy attenuation rate. $s_{H}x\left(T\right)e^{-rT}$ and $s_{L}x\left(T\right)e^{-rT}$ are the terminal cost for the two categories of wireless nodes. Based on (3), we can find the optimal solutions for information transmission, which are mainly varied based on the unit price of transferred energy. After the UAV announces the unit energy price for the two categories, the wireless nodes can make optimal decisions. The unit price of the transferred energy is based on the optimal solution of (5) and (6), combined with (4).

## 4. Optimal Solutions for Energy Transfer

In this section, we discuss the solutions to the resource allocation problem given in (5) and (6). The UAV is considered to be the leader in the proposed dynamic game. The UAV should achieve optimal power control for energy transfer based on (5) and (6), and the optimal unit energy price can also be achieved. In order to solve the above problems, a feedback Nash equilibrium can be characterized as follows.

**Definition 1.**  For each nodes category, there exists optimal feedback solutions, which are denoted by $\left\{\pi_{k}^{*}\left(t\right),p_{k}^{*}\left(t\right)\right\}\left(k=\left\{H,L\right\}\right)$, if continuously differentiable functions $V^{k}\left(t,x\right)$ exist for each nodes category k={H,L}, and $V^{k}\left(t,x\right)$ should satisfy the following differential equations [29]:

(7) $$-V_{t}^{H}\left(t,x\right)=\underset{\pi_{H}^{}\left(t\right),p_{H}^{}\left(t\right)}{\max}\left\{\begin{array}{l} \left[\begin{array}{l} \sum_{i=1}^{N_{H}}\left[{\left[\gamma_{H,i}\left(p_{H,i}\left(t\right)\right)-\gamma_{th}\right]}^{2}-\pi_{H}\left(t\right)p_{H,i}\left(t\right)\right]-c_{H}p_{H}^{2}\left(t\right)\\ -\varepsilon_{H}^{H}\sum_{i=1}^{N_{H}}p_{H,i}\left(t\right)-\varepsilon_{H}^{L}\sum_{i=1}^{N_{L}}p_{L,i}\left(t\right)-\mu_{H}x\left(t\right) \end{array}\right]e^{-rt}\\ +V_{x}^{H}\left(t,x\right)\left[\sum_{k=\left\{H,L\right\}}^{}\eta_{k}p_{k}\left(t\right)+\sum_{i=1}^{N_{H}}p_{H,i}\left(t\right)+\sum_{i=1}^{N_{L}}p_{L,i}\left(t\right)+\delta x\left(t\right)\right] \end{array}\right\}$$

(8) $$V^{H}\left(T,x\right)=s_{H}x\left(T\right)e^{-rT}$$

(9) $$-V_{t}^{L}\left(t,x\right)=\underset{\pi_{L}^{}\left(t\right),p_{L}^{}\left(t\right)}{\max}\left\{\begin{array}{l} \left[\begin{array}{l} \sum_{i=1}^{N_{L}}\left[{\left[\gamma_{L,i}\left(p_{L,i}\left(t\right)\right)-\gamma_{th}\right]}^{2}-\pi_{L}\left(t\right)p_{L,i}\left(t\right)\right]-c_{L}p_{L}^{2}\left(t\right)\\ -\varepsilon_{L}^{L}\sum_{i=1}^{N_{L}}p_{L,i}\left(t\right)-\varepsilon_{L}^{H}\sum_{i=1}^{N_{H}}p_{H,i}\left(t\right)-\mu_{L}x\left(t\right) \end{array}\right]e^{-rt}\\ +V_{x}^{L}\left(t,x\right)\left[\sum_{k=\left\{H,L\right\}}^{}\eta_{k}p_{k}\left(t\right)+\sum_{i=1}^{N_{H}}p_{H,i}\left(t\right)+\sum_{i=1}^{N_{L}}p_{L,i}\left(t\right)+\delta x\left(t\right)\right] \end{array}\right\}$$

(10) $$V^{L}\left(T,x\right)=s_{L}x\left(T\right)e^{-rT},$$

*where,*

(11) $$V^{H}\left(t\right)=\max\int_{0}^{T}\left[\begin{array}{l} \sum_{i=1}^{N_{H}}\left[{\left[\gamma_{H,i}\left(p_{H,i}\left(t\right)\right)-\gamma_{th}\right]}^{2}-\pi_{H}\left(t\right)p_{H,i}\left(t\right)\right]-c_{H}p_{H}^{2}\left(t\right)\\ -\varepsilon_{H}^{H}\sum_{i=1}^{N_{H}}p_{H,i}\left(t\right)-\varepsilon_{H}^{L}\sum_{i=1}^{N_{L}}p_{L,i}\left(t\right)-\mu_{H}x\left(t\right) \end{array}\right]e^{-rt}dt+s_{H}x\left(T\right)e^{-rT},$$

(12) $$V^{L}\left(t\right)=\max\int_{0}^{T}\left[\begin{array}{l} \sum_{i=1}^{N_{L}}\left[{\left[\gamma_{L,i}\left(p_{L,i}\left(t\right)\right)-\gamma_{th}\right]}^{2}-\pi_{L}\left(t\right)p_{L,i}\left(t\right)\right]-c_{L}p_{L}^{2}\left(t\right)\\ -\varepsilon_{L}^{L}\sum_{i=1}^{N_{L}}p_{L,i}\left(t\right)-\varepsilon_{L}^{H}\sum_{i=1}^{N_{H}}p_{H,i}\left(t\right)-\mu_{L}x\left(t\right) \end{array}\right]e^{-rt}dt+s_{L}x\left(T\right)e^{-rT}$$

In the above equations, the functions $V^{k}\left(t,x\right)$ are the objective functions of the UAV for profit maximization during the wireless energy transfer for the two categories. The observation time for the profit maximization is set to be [0,T]. The UAV can control its power transfer level for the two categories of wireless nodes based on the objective functions during the observation time [0,T]. Substituting Formulas (1) and (3) into Formulas (7) and (9), we can obtain the following differential equations:

(13) $$-V_{t}^{H}\left(t,x\right)=\underset{\pi_{H}^{}\left(t\right),p_{H}^{}\left(t\right)}{\max}\left\{\begin{array}{l} \left[\begin{array}{l} \sum_{i=1}^{N_{H}}\left[-\frac{1}{4}\pi_{H}^{2}\left(t\right)\frac{I_{H,i}^{2}\left(t\right)}{g_{H,i}^{2}\left(t\right)}-\pi_{H}\left(t\right)\gamma_{th}\frac{I_{H,i}^{}\left(t\right)}{g_{H,i}^{}\left(t\right)}\right]-c_{H}p_{H}^{2}\left(t\right)\\ -\varepsilon_{H}^{H}\sum_{i=1}^{N_{H}}\left(\frac{1}{2}\pi_{H}\left(t\right)\frac{I_{H,i}^{2}\left(t\right)}{g_{H,i}^{2}\left(t\right)}+\gamma_{th}\frac{I_{H,i}^{}\left(t\right)}{g_{H,i}^{}\left(t\right)}\right)\\ -\varepsilon_{H}^{L}\sum_{i=1}^{N_{L}}\left(\frac{1}{2}\pi_{L}\left(t\right)\frac{I_{L,i}^{2}\left(t\right)}{g_{L,i}^{2}\left(t\right)}+\gamma_{th}\frac{I_{L,i}^{}\left(t\right)}{g_{L,i}^{}\left(t\right)}\right)-\mu_{H}x\left(t\right) \end{array}\right]e^{-rt}\\ +V_{x}^{H}\left(t,x\right)\left[\begin{array}{l} \sum_{k=\left\{H,L\right\}}^{}\eta_{k}p_{k}\left(t\right)+\sum_{i=1}^{N_{H}}\left(\frac{1}{2}\pi_{H}\left(t\right)\frac{I_{H,i}^{2}\left(t\right)}{g_{H,i}^{2}\left(t\right)}+\gamma_{th}\frac{I_{H,i}^{}\left(t\right)}{g_{H,i}^{}\left(t\right)}\right)\\ +\sum_{i=1}^{N_{L}}\left(\frac{1}{2}\pi_{L}\left(t\right)\frac{I_{L,i}^{2}\left(t\right)}{g_{L,i}^{2}\left(t\right)}+\gamma_{th}\frac{I_{L,i}^{}\left(t\right)}{g_{L,i}^{}\left(t\right)}\right)+\delta x\left(t\right) \end{array}\right] \end{array}\right\},$$

(14) $$-V_{t}^{L}\left(t,x\right)=\underset{\pi_{L}^{}\left(t\right),p_{L}^{}\left(t\right)}{\max}\left\{\begin{array}{l} \left[\begin{array}{l} \sum_{i=1}^{N_{L}}\left[-\frac{1}{4}\pi_{L}^{2}\left(t\right)\frac{I_{L,i}^{2}\left(t\right)}{g_{L,i}^{2}\left(t\right)}-\pi_{L}\left(t\right)\gamma_{th}\frac{I_{L,i}^{}\left(t\right)}{g_{L,i}^{}\left(t\right)}\right]-c_{L}p_{L}^{2}\left(t\right)\\ -\varepsilon_{L}^{L}\sum_{i=1}^{N_{L}}\left(\frac{1}{2}\pi_{L}\left(t\right)\frac{I_{L,i}^{2}\left(t\right)}{g_{L,i}^{2}\left(t\right)}+\gamma_{th}\frac{I_{L,i}^{}\left(t\right)}{g_{L,i}^{}\left(t\right)}\right)\\ -\varepsilon_{L}^{H}\sum_{i=1}^{N_{H}}\left(\frac{1}{2}\pi_{H}\left(t\right)\frac{I_{H,i}^{2}\left(t\right)}{g_{H,i}^{2}\left(t\right)}+\gamma_{th}\frac{I_{H,i}^{}\left(t\right)}{g_{H,i}^{}\left(t\right)}\right)-\mu_{L}x\left(t\right) \end{array}\right]e^{-rt}\\ +V_{x}^{L}\left(t,x\right)\left[\begin{array}{l} \sum_{k=\left\{H,L\right\}}^{}\eta_{k}p_{k}\left(t\right)+\sum_{i=1}^{N_{H}}\left(\frac{1}{2}\pi_{H}\left(t\right)\frac{I_{H,i}^{2}\left(t\right)}{g_{H,i}^{2}\left(t\right)}+\gamma_{th}\frac{I_{H,i}^{}\left(t\right)}{g_{H,i}^{}\left(t\right)}\right)\\ +\sum_{i=1}^{N_{L}}\left(\frac{1}{2}\pi_{L}\left(t\right)\frac{I_{L,i}^{2}\left(t\right)}{g_{L,i}^{2}\left(t\right)}+\gamma_{th}\frac{I_{L,i}^{}\left(t\right)}{g_{L,i}^{}\left(t\right)}\right)+\delta x\left(t\right) \end{array}\right] \end{array}\right\}$$

Calculating the partial derivative for $\pi_{H}^{}\left(t\right)$ and $\pi_{L}^{}\left(t\right)$ in (13) and (14), the optimal unit price for the wireless power transfer can be given as follows:

(15) $$\pi_{H}^{*}\left(t\right)=V_{x}^{H}\left(t,x\right)e^{rt}-\varepsilon_{H}^{H}-2\gamma_{th}\sum_{i=1}^{N_{H}}\frac{g_{H,i}^{}\left(t\right)}{I_{H,i}^{}\left(t\right)}+\sum_{j=1,j\neq i}^{N_{H}}\frac{I_{H,j}^{2}\left(t\right)}{g_{H,j}^{2}\left(t\right)}\cdot \frac{g_{H,i}^{2}\left(t\right)}{I_{H,i}^{2}\left(t\right)}\left[V_{x}^{H}\left(t,x\right)e^{rt}-\varepsilon_{H}^{H}\right],$$

(16) $$\pi_{L}^{*}\left(t\right)=V_{x}^{L}\left(t,x\right)e^{rt}-\varepsilon_{L}^{L}-2\gamma_{th}\sum_{i=1}^{N_{L}}\frac{g_{L,i}^{}\left(t\right)}{I_{L,i}^{}\left(t\right)}+\sum_{j=1,j\neq i}^{N_{L}}\frac{I_{L,j}^{2}\left(t\right)}{g_{L,j}^{2}\left(t\right)}\cdot \frac{g_{L,i}^{2}\left(t\right)}{I_{L,i}^{2}\left(t\right)}\left[V_{x}^{L}\left(t,x\right)e^{rt}-\varepsilon_{L}^{L}\right].$$

Meanwhile, solving (13) and (14), the optimal allocated resource for wireless energy transfer $p_{H}^{}\left(t\right)$ and $p_{L}^{}\left(t\right)$ can be given as follows:

(17) $$p_{H}^{*}\left(t\right)=\frac{\eta_{H}V_{x}^{H}\left(t,x\right)e^{rt}}{2c_{H}},$$

(18) $$p_{L}^{*}\left(t\right)=\frac{\eta_{L}V_{x}^{L}\left(t,x\right)e^{rt}}{2c_{L}}.$$

**Theorem 1.**  *The value functions $V^{H}\left(t,x\right)$ and $V^{L}\left(t,x\right)$ given in (11) and (12) can be obtained as follows:*

(19) $$V^{H}\left(t,x\right)=\left[A_{H}\left(t\right)x+B_{H}\left(t\right)\right]e^{-rt},$$

(20) $$V^{L}\left(t,x\right)=\left[A_{L}\left(t\right)x+B_{L}\left(t\right)\right]e^{-rt},$$

*where $A_{H}\left(t\right)$ is given by*

(21) $$A_{H}\left(t\right)=\left(\frac{\mu_{H}}{r-\delta }+s_{H}\right)e^{\left(r-\delta \right)\left(t-T\right)}-\frac{\mu_{H}}{r-\delta },$$

(22) $$A_{H}\left(T\right)=s_{H},$$

*and $A_{L}\left(t\right)$ is given by*

(23) $$A_{L}\left(t\right)=\left(\frac{\mu_{L}}{r-\delta }+s_{L}\right)e^{\left(r-\delta \right)\left(t-T\right)}-\frac{\mu_{L}}{r-\delta },$$

(24) $$A_{L}\left(T\right)=s_{L}.$$

**Proof.**  By taking the derivative of $V^{H}\left(t,x\right)$ with respect to t and x, we obtain

(25) $$V_{t}^{H}\left(t,x\right)=\left\{\left[-rA_{H}\left(t\right)+{\dot{A}}_{H}\left(t\right)\right]x+\left[-rB_{H}\left(t\right)+{\dot{B}}_{H}\left(t\right)\right]\right\}e^{-rt},$$

(26) $$V_{x}^{H}\left(t,x\right)=A_{H}\left(t\right)e^{-rt}.$$

Substituting (25) and (26) into (13), $A_{H}^{}\left(t\right)$ is satisfied:

(27) $${\dot{A}}_{H}\left(t\right)=\left(r-\delta \right)A_{H}\left(t\right)+\mu_{H}.$$

Then, we obtain

(28) $$A_{H}\left(t\right)=\left(\frac{\mu_{H}}{r-\delta }+s_{H}\right)e^{\left(r-\delta \right)\left(t-T\right)}-\frac{\mu_{H}}{r-\delta }.$$

Similarly, we can obtain

(29) $$A_{L}\left(t\right)=\left(\frac{\mu_{L}}{r-\delta }+s_{L}\right)e^{\left(r-\delta \right)\left(t-T\right)}-\frac{\mu_{L}}{r-\delta }.$$

Based on (28) and (29), the optimal allocated resource in (17) and (18), which denotes the UAV’s strategies for wireless energy transfer, can be re-written as follows:

(30) $$p_{H}^{*}\left(t\right)=\frac{\eta_{H}}{2c_{H}}\left[\left(\frac{\mu_{H}}{r-\delta }+s_{H}\right)e^{\left(r-\delta \right)\left(t-T\right)}-\frac{\mu_{H}}{r-\delta }\right],$$

(31) $$p_{L}^{*}\left(t\right)=\frac{\eta_{L}}{2c_{L}}\left[\left(\frac{\mu_{L}}{r-\delta }+s_{L}\right)e^{\left(r-\delta \right)\left(t-T\right)}-\frac{\mu_{L}}{r-\delta }\right]$$

Meanwhile, we can obtain the optimal unit price for the wireless power transfer as follows:

(32) $$\begin{array}{l} \pi_{H}^{*}\left(t\right)=\left(\frac{\mu_{H}}{r-\delta }+s_{H}\right)e^{\left(r-\delta \right)\left(t-T\right)}-\frac{\mu_{H}}{r-\delta }-\varepsilon_{H}^{H}-2\gamma_{th}\sum_{i=1}^{N_{H}}\frac{g_{H,i}^{}\left(t\right)}{I_{H,i}^{}\left(t\right)}\\ +\sum_{j=1,j\neq i}^{N_{H}}\frac{I_{H,j}^{2}\left(t\right)}{g_{H,j}^{2}\left(t\right)}\cdot \frac{g_{H,i}^{2}\left(t\right)}{I_{H,i}^{2}\left(t\right)}\left[\left(\frac{\mu_{H}}{r-\delta }+s_{H}\right)e^{\left(r-\delta \right)\left(t-T\right)}-\frac{\mu_{H}}{r-\delta }-\varepsilon_{H}^{H}\right] \end{array}$$

(33) $$\begin{array}{l} \pi_{L}^{*}\left(t\right)=\left(\frac{\mu_{L}}{r-\delta }+s_{L}\right)e^{\left(r-\delta \right)\left(t-T\right)}-\frac{\mu_{L}}{r-\delta }-\varepsilon_{L}^{L}-2\gamma_{th}\sum_{i=1}^{N_{L}}\frac{g_{L,i}^{}\left(t\right)}{I_{L,i}^{}\left(t\right)}\\ +\sum_{j=1,j\neq i}^{N_{L}}\frac{I_{L,j}^{2}\left(t\right)}{g_{L,j}^{2}\left(t\right)}\cdot \frac{g_{L,i}^{2}\left(t\right)}{I_{L,i}^{2}\left(t\right)}\left[\left(\frac{\mu_{L}}{r-\delta }+s_{L}\right)e^{\left(r-\delta \right)\left(t-T\right)}-\frac{\mu_{L}}{r-\delta }-\varepsilon_{L}^{L}\right] \end{array}$$

Based on the process used to obtain the optimal solutions for the energy transfer, we find that the time complexity of the algorithm will be $O\left(n^{2}\right)$. In the proposed game model, the optimal solutions for energy transfer should be solved for the wireless nodes in each group, which should also be calculated for each time point in a finite time horizon. Then, the time complexity of the algorithm should be $O\left(n^{2}\right)$. □

## 5. Numerical Simulations

**Figure 3:**
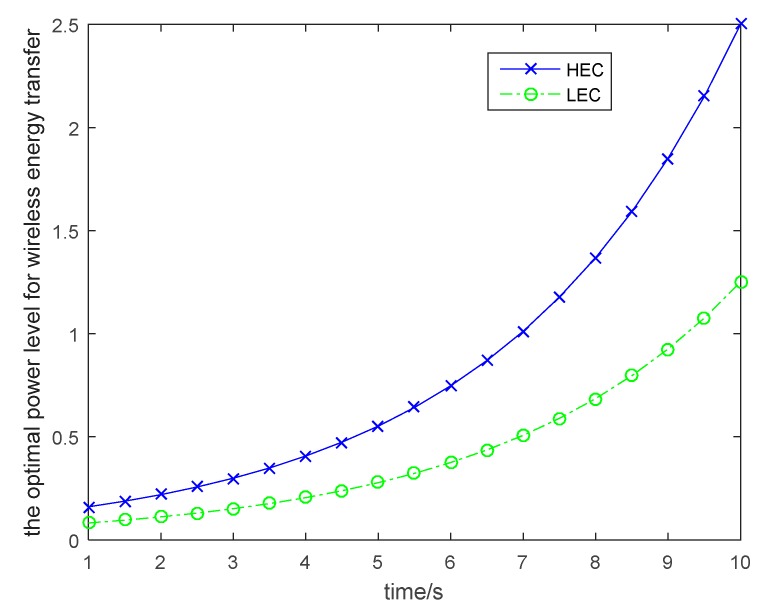
Power level for wireless energy transfer.

In this section, we will simulate the proposed dynamic game to get the optimal allocated resource level for the UAV. MATLAB software is used to construct the simulation environment. The simulated IoT system includes two kinds of wireless nodes, which have different energy consumption requirements. Based on the Nash equilibrium given in Section 4, we can get the optimal power level of wireless energy transfer for each kind of wireless node. Figure 3 shows the optimal allocated resources of wireless energy transfer for each kind of wireless node. The UAV should transmit the energy to the wireless nodes based on the nodes’ requirements and maximize the profit during the energy transfer. As shown in the Figure 3, the wireless nodes in the HEC group need more energy compared to the wireless nodes in the LEC group, because the energy consumption in the HEC group is higher than that in the LEC group. As the game continues, the UAV needs to increase its power level for wireless energy transfer to satisfy more requirements of the wireless nodes, and to increase its profit.

**Figure 4:**
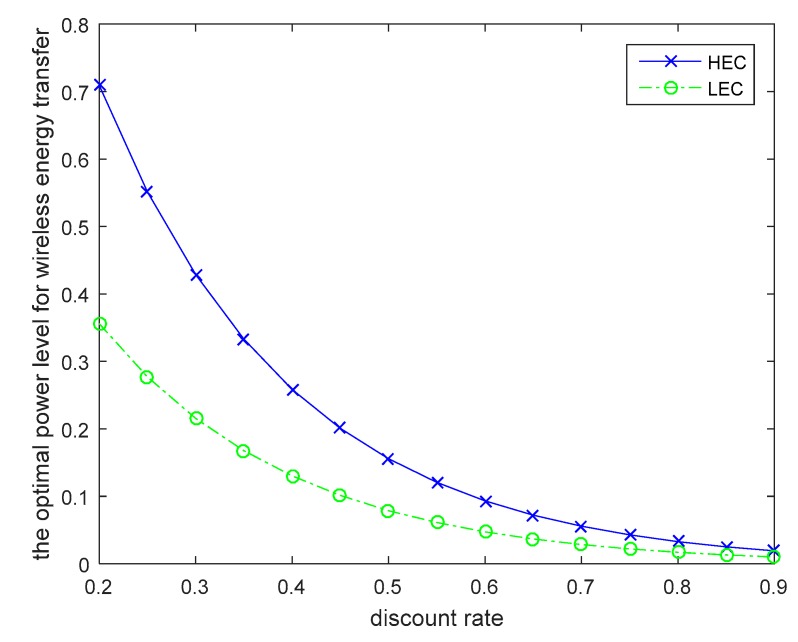
Power level for wireless energy transfer with different discount rates.

**Figure 5:**
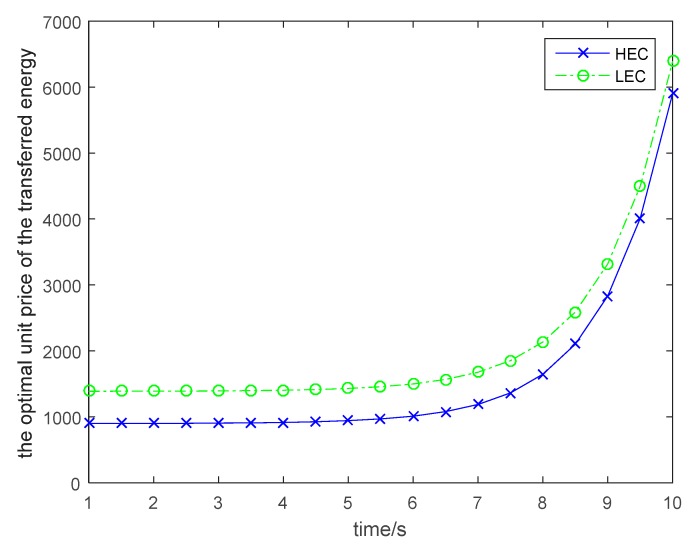
Optimal unit price of the transferred energy.

**Figure 6:**
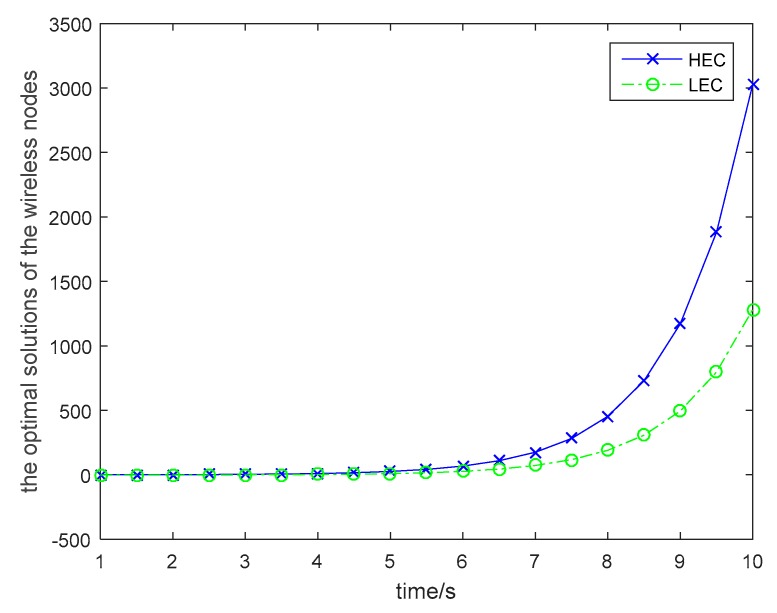
Optimal solutions of the wireless nodes.

The discount factor also affects the optimal policies for wireless energy transfer, which is shown in Figure 4. As shown in Figure 4, the optimal power level for wireless energy transfer is decreased with the incensement of the discount rate. The optimal unit price for the transferred energy is given in Figure 5. With the time incensement, the UAV will increase the unit price for the transferred energy, because the larger the energy transfer time, the higher the transfer cost for the UAV. As the energy conversion efficiency is low for wireless energy transfer, and it is a long distance for wireless energy transfer, the UAV should carry more energy for wireless power transfer. All these factors will cause an exponential increase in the energy transfer cost. Then, the UAV should increase the unit price for the transferred energy when the time required for the wireless power transfer is large. In Figure 6, the optimal solutions for the wireless nodes are given. In order to simplify the simulations for the users, we assume that all wireless nodes in the same category are uniform and standard. From Figure 6, we can find that the wireless sensors will increase the transmitted power for information transmission to earn more profit, although the unit price for the transferred energy is increased over time. Based on Figure 3, we can see that the wireless nodes will have more energy over time, because the UAV increases its power level for wireless energy transfer to satisfy more requirements of the wireless nodes. Then the wireless nodes can harvest more energy and will have more energy for information transmission.

## 6. Conclusions

In this paper, we have researched the resource allocation problems in a UAV-assisted IoT system based on a dynamic game. In the proposed model, the wireless nodes are divided into two categories for energy harvesting. Each node category has its own power level transferred from the UAV. The UAV can control its power for energy transfer based on the proposed dynamic game. Meanwhile, as the harvested energy of the wireless nodes is mainly controlled by the unit energy price, the optimal unit price of the transferred energy for the two categories of nodes can also obtained based on the proposed dynamic game model. In order to get the optimal solutions for the resource allocation problems, we use dynamic programming to obtain the Nash equilibriums of the proposed dynamic game. The simulation results show that the proposed game model can achieve optimal power control and price control for the UAV.
